# Biocultural intersections: stressors, adaptability, allostasis, frailty, and aging

**DOI:** 10.1186/s40101-022-00307-y

**Published:** 2022-09-24

**Authors:** Douglas Crews

**Affiliations:** The Ohio State University, Columbus, USA

Papers in this collection address multiple research areas within human physiological, biological, and biocultural anthropology. Applying ecological and evolutionary theory, physiological anthropologists explore and document influences of environmental, biocultural, and social stressors on human variation across the life span by sex, location, and migratory status within and among groups. Using biocultural perspectives, each paper explores ecological, bioethnographic, and/or life history factors to understand variable human responses to stressors across environmental and sociocultural settings. Jointly, they address responses to climate, migration, aging, reproduction, frailty, and human adaptability. Applying variable research methods and data acquisition techniques and ranging from a literature review to empirically focused research, these papers share a common thread, exploring human adaptability in response to stressful environments as a biocultural process.

Included are a bioethnographic exploration of stress, allostatic load, and perceived health among migrants from Mexico to the USA, frailty as a possible adjunct to activities of daily living and other scales when designing senior housing, influences of reproductive histories on frailty among US and Philippine women, and assessing the thermoregulatory physiology of brown adipose tissue and its importance for maintaining body temperature in a subarctic setting. Each paper addresses human physiological variation as a response to variable environmental and sociocultural settings, lifeways, population, and individual histories, all current issues in physiological anthropology and human biology. These papers include stress, migration, and allostatic load, a model based on Mexican migrants in Columbus, Ohio [1]; aging, frailty, and design of built environments [2]; brown adipose tissue thermogenesis among a small sample of reindeer herders from subarctic Finland [3]; and women’s lifetime reproductive profiles and frailty among aging populations in the USA and the Philippines [4].

Tuggle et al. [1] developed a multifactor model for assessing stress among migrants using a sample of immigrants to Columbus and Central Ohio from Mexico. Their contribution introduces and illustrates multiple theories, topics, and concepts revisited in the three additional papers. Based in ecological theory, Tuggle and colleagues [1] implemented a biocultural/ethnographic research design. They included face-to-face interviews, self-report health questionnaires and health narratives, and a battery of quantitative physiological assessments of health and allostasis. Using these multiple data types, they explored congruence between how migrants self-reported their stressor exposures, health, well-being, and happiness and their assessed health. Results suggest, neither personal narratives nor self-reports of health significantly predict quantitative assessments of health, indicating these domains of health, self-reported and physiologically assessed, may vary independently. Tuggle et al. [1] suggest future research will require mixed methods and a bioethnographic approach to understand physiological and culturally embedded processes related to migration and health. Physiological stressor responses reflect multiple biological and physiological systems intersecting with sociocultural and environmental variability. Fully understanding and unraveling these complex multifactorial relationships occurring among migration, acculturation, and health likely will require incorporating individual health narratives, along with self-reports, and assessments of physiological biomarkers. They conclude assessing physiological biomarkers along with self-reports and narratives provides a more complex web of interactions for connecting intersecting pathways influencing health and risks for ill-health.

In the second contribution, Crews [2] suggests human variation and frailty provide useful criteria for improving designs of built environments and physical settings for congregate senior living. To begin, the concept of frailty, its measurement and prevalence among seniors, along with physical and sensory losses accompanying older age are detailed. Next, Crews [2] reviews briefly congregate senior housing designs from the early through the mid-twentieth century, along with latter innovative design improvements providing more supportive and accommodating environments. Included are examples of positive and negative physiological responses to variable lighting schemes, floor plans, acoustics, furnishings, and mechanicals. The reviewer suggests collaborations between architects and physiological anthropologists in designing built environments for seniors will promote insights for improving accessibility and accommodating seniors’ frailty and ability limitations. Interior and exterior designs specific to senior’s physical and physiological capabilities and limitations will better support them in maintaining accessibility to their built environments. Crews [1] suggests collaborations among physiological, biocultural, and cultural anthropologists along and with architects, designers, and builders will aid in improving designs of built environments for elder care and housing. Traditional anthropological methods, participant observation, interviews, consensus analysis, narratives with all clients (e.g., residents, staff, administrators, funders, boards of trustees), anthropometry, and physiological assessments are all applicable to designing environments for seniors. Jointly, these activities should expand our understanding of how structural designs may support or constrain accessibility by seniors.

The third report included herein is Ocobock and colleagues’ [3] research on brown adipose tissue (BAT)-responsive thermogenesis in a sample of subarctic Finland reindeer herders. BAT is a topic of perennial interest in human biology and physiological and biological anthropology. Ocobock et al. [3] begin with a brief review of human thermic response physiology, focusing their attention on BAT’s thermoregulatory role in maintaining core temperature. They suggest BAT responsiveness to whole-body cooling maintains basic core temperature among cold-stressed populations. To test their hypothesis, they enrolled 22 cold-climate adapted reindeer herders (6 females/16 males) and monitored their metabolic rates and surface temperatures during mild cold exposure. Using indirect calorimetry and thermal imaging of BAT-positive and BAT-negative body regions, participants were exposed to ambient room temperature (20–27 °C) and mild cold exposure (15–18 °C). Among these Finnish cold-climate adapted reindeer herders, average metabolic rate increased during mild cold exposure. They showed significantly warmer surface temperatures over their BAT-positive regions compared to BAT-negative ones. These results support a model that cold-adapted herders activate BAT thermogenesis during mild cold exposure. Furthermore, they preferentially metabolize lipids to support this elevated body temperature. Ocobock et al. [3] propose BAT thermogenesis is a physiological response to body cooling and a likely key component in maintaining body temperature across cold-stressed populations.

In the final contribution, Dorne and Zoorob [4] explore associations between lifetime reproductive profiles and frailty among postreproductive women in the USA and the Philippines. Basing their research on life history theory suggesting trade-offs between investments in somatic survival and reproduction, Dorne and Zoorob [4] hypothesize a trade-off between health in later life, determined by biomarkers of frailty, and number of offspring ever born to postmenopausal women. They suggest a trade-off occurs as resources invested in reproducing and fledging offspring reduce somatic resources remaining to support longer lives of mothers due to antagonistic pleiotropy. An alternative model suggests pregnancy, along with lactation over multiple years, benefits women’s survival: i.e., more children born and more time breastfeeding promote health and survival. Dorne and Zoorob [4] estimate frailty by assessing strength, walking speed, standing ability, and mobility. They suggest cumulative alterations in and ebbs and flows of steroid hormones over a lifetime, total lengths of gestations, and time lactating are less detrimental to women’s health than are cumulative ebbs and flows of steroid hormones during menstrual cycling. For example, estradiol may be beneficial during reproductive years but detrimental to later life survival. Dorne and Zoorob [4] hypothesize fewer lifetime menstrual cycles (measured as more children, more pregnancies, and higher frequencies of breastfeeding) during reproductive years will associate significantly with higher frailty during post-reproductive years. Samples of women aged 65+ were recruited from the Philippines (*N* = 67, average age 73.0) and USA (*N* = 44, average age = 69.4). Frailty was assessed as grip strength, gait speed, and the sit-to-stand exercise. Menstrual cycles were assessed by number of months not pregnant or lactating. Estimated lifetime cycles (ELC) failed to associate significantly with frailty assessments or overall health. ELC did associate significantly with BMI. Furthermore, while ELC did not associate significantly with frailty in the US sample, among the Philippine women, higher ELC trended toward being associated with lower frailty. No estimate of frailty differed significantly between high and low ELC subgroups within samples. They suggest other factors, activity levels, health parameters, or exogenous exposure to sex steroids may influence lifetime sex steroids and frailty, as may additional factors not assayed, e.g., activity patterns, type of labor, or physical limitations.

Papers in this collection integrate physiological, social, cultural, and environmental variability as a framework to assess quantitative and qualitative aspects of health, frailty, allostatic load, and thermal response. Each applies a multifaceted biocultural and bioethnographic model of human adaptability and variation as responses to environmental and ecological stressors. By assessing and contextualizing environmental, social, and cultural influences on human physiological, functional, and physical variability, these human biologists and physiological and biological anthropologists were able to develop and test holistic models of human health, well-being, and responses to stressors across populations.
